# Post-COVID-19 Psychiatric Symptoms in the Elderly: The Role of Gender and Resilience

**DOI:** 10.3390/jpm12122016

**Published:** 2022-12-06

**Authors:** Delfina Janiri, Matteo Tosato, Alessio Simonetti, Silvia Montanari, Beatrice Terenzi, Antonello Catinari, Lorenzo De Mori, Gaspare Filippo Ferrajoli, Georgios D. Kotzalidis, Francesco Landi, Roberto Bernabei, Gabriele Sani

**Affiliations:** 1 Department of Neuroscience, Section of Psychiatry, Università Cattolica del Sacro Cuore, Largo Francesco Vito 1, 00168 Rome, Italy; 2 Department of Psychiatry and Neurology, Sapienza University of Rome, Piazzale Aldo Moro 5, 00185 Rome, Italy; 3 Department of Psychiatry, Fondazione Policlinico Universitario Agostino Gemelli IRCCS, Largo Francesco Vito 1, 00168 Rome, Italy; 4 Geriatrics Department, Fondazione Policlinico Universitario Agostino Gemelli IRCCS, Largo Francesco Vito 1, 00168 Rome, Italy; 5 Department of Neurosciences, Mental Health and Sensory Organs (NESMOS), Sapienza University of Rome, Via di Grottarossa 1035-1039, 00189 Rome, Italy

**Keywords:** COVID-19, depression, anxiety, gender, women, resilience, psychiatric symptoms

## Abstract

COVID-19 represents an overwhelming stressor to mental health. Elderly individuals are particularly at risk, but it is still unclear whether the risk is equally distributed among men and women. The aim of this study was to define gender differences in persistent psychiatric symptoms after COVID-19 illness and to test their association with resilience factors. **Methods:** We assessed 348 individuals aged >65 years at a multidisciplinary post-COVID-19 service. Mood and anxiety symptoms were investigated, as well as psychological distress and resilience, as assessed with the Kessler-10 (K10) Scale and the Connor-Davidson Resilience Scale (CD-RISC), respectively. Multivariate and linear regression analyses were used to test the distribution patterns of psychiatric symptoms and resilience factors. **Results:** In the total sample, 214 (61.5%) were men (a mean age of 73.25 years ±6.04) and 134 (38.5%) were women (a mean age of 72.69 years ±6.43; *p* = 0.407). Men and women significantly differed in marital status (*χ*^2^ = 25.17; *p* < 0.001, more men were married), living alone (*χ*^2^ = 11.62; *p* < 0.01, fewer men were living alone), hospitalization during COVID-19 illness (*χ*^2^ = 12.35; *p* < 0.001, more men were hospitalized during COVID-19), and subjective health status before COVID-19 infection (*χ*^2^ = 4.32; *p* < 0.001, men reporting better subjective health than women). Women reported more psychiatric symptoms and fewer resilience factors than men. Low resilience levels significantly predicted psychological distress in both men and women. **Conclusions:** The female elderly population affected by COVID-19 showed a greater vulnerability to psychiatric symptoms. Our data point to the need to strengthen resilience resources, especially in women.

## 1. Introduction

The coronavirus (COVID-19) pandemic represents an overwhelming stressor to mental health. The COVID-19 outbreak was associated with various psychological problems in the general population and with an increased risk of developing psychiatric symptoms [1] or exacerbating them [2,3]. Patients who developed COVID-19 are particularly at risk [4]. Individuals who experienced severe acute COVID-19 illness reported a high prevalence of persistent psychiatric symptoms. Specifically, a large meta-analysis reported depressed mood and anxiety among the most frequent complaints in the post-illness stage [5].

Elderly individuals have been identified as a particularly fragile population in terms of mental health. Over 20% of elderly individuals suffer from a mental or neurological disorder, which accounts for 6.6% of the total disability (DALYs) for this age group [6]. Depression and anxiety are the most common mental illnesses, affecting approximately 7% and 3.8%, respectively, of the world’s aged population, according to World Health Organization (WHO) data. The effect of the COVID-19 pandemic on this population has been prominent [7,8]. In particular, in a recent study, our group demonstrated that having had COVID-19 is related to a high likelihood of psychological distress in advanced-age people [9]. Nevertheless, the role of individual factors in determining patients’ likelihood to develop psychiatric symptoms needs further investigation. In particular, it is still unclear whether the risk in the elderly is equally distributed between men and women. The literature mentions the importance of disaggregating findings gender-wise, highlighting that population subgroups have different risks and outcomes and that interventions need to be accordingly patient-tailored [10].

Previous studies in middle-aged COVID-19 survivors focused on gender differences in experiencing adverse psychological outcomes and neuropsychiatric complications [11], finding that women were more at risk than men. In parallel, a recent study explored gender in psychological resilience following the COVID-19 pandemic in older adults during the first lockdown in Italy. Authors found that older women might appear to be more vulnerable in facing the pandemic, compared to men. These observations need to be confirmed in the elderly COVID-19 survivors [12].

Emotional responses to biological threats may vary with time and with specific conditions [13,14]. Psychological responses to COVID-19 depend on what the average people believe about COVID-19, and their views may vary across the various waves of the pandemic. However, longitudinal studies involving repeat assessments throughout the COVID-19 waves yielded somehow stable emotional responses regarding anxiety and depression [15,16,17]. So, given the overall emotional stability found in the literature, we decided to assess distress, mood, anxiety, and resilience in a sample of aged people who had recovered from COVID-19 cross-sectionally in the first moment.

In line with the above observations, we investigated sex differences in persistent psychiatric symptoms in a large cohort of patients aged >65 years who contracted the infection and recovered from COVID-19 illness. We capitalized on the availability of data from a multidisciplinary post-acute care service established in the early phase of the COVID-19 outbreak in Rome, Italy. A secondary aim was the relationship between the risk of developing psychiatric symptoms and individual resilience factors, in both men and women participants. We predicted that positive personal attributes for resilience may be protective against psychopathological risk.

## 2. Methods

### 2.1. Patient Sample

Patients aged >65 years who had contracted a COVID-19 infection from April 2020 to October 2021 and recovered from the infection were included in this study. Eligible patients were sought from those referring to the multidisciplinary post-acute care service, the workplace of multiple specialists, that has been established at the Fondazione Policlinico Universitario Agostino Gemelli IRCCS, Università Cattolica del Sacro Cuore of Rome (Rome, Italy) [18]. Patients, after their discharge, had to test negative for two consecutive nasal and/or nasopharyngeal swabs and be fever-free and should have been referred to the post-COVID-19 service. They all volunteered to participate. Those unable to provide informed consent or were not sufficiently fluent in Italian to be able to complete the questionnaires were excluded. Other exclusion criteria were severe neuropsychiatric disorders such as dementia, delusional and psychotic disorders, and neurodevelopmental disorders that could interfere with the completion of questionnaires. A total of 348 patients were enrolled in the study. The assessment was comprehensive and included medical and psychiatric history, physical examination, and psychiatric status. It took place 30–120 days after recovery for all patients. Demographic and epidemiological characteristics, including clinical and drug treatment history, were investigated and inserted in a database. Further information regarding patient recruitment may be found in previous studies [9].

### 2.2. Assessment

The mood was explored using the Hamilton Depression Rating Scale (HDRS) and the Young Mania Rating Scale (YMRS). The **HDRS** [19] is considered the gold standard for assessing the severity of depression, as it is sensitive to change and able to assess the effect of treatment on patients. The HDRS has several versions, with the number of items employed ranging from 17 to 28, but only the first 17 items are included in computations. Each of the behaviorally anchored items is rated by the clinician on either a 3- or 5-point Likert scale and summed to obtain the total score. Scores of ≤7 are considered as having no depression, while ≥8 define the range of depression of variable severity. Mild depression is defined by a score of 8–16, moderate depression by a score of 17–23, and severe depression is any score of ≥24. Within the scale, we also used the subgroup of items that describe specifically somatic symptoms as a separate score.

The **YMRS** [20] is a clinician-administered scale that explores the key symptoms of mania on the basis of what patients report about their condition in the last 48 h and the clinician’s observation of the patient’s behavior during the interview, with the latter being more important than the former for the final assessment. The scale consists of 11 items that investigate mood, motor activity, quantitative and formal thought disorders, illness awareness, aggression, libido, sleep, and behavioral disturbances. The higher the scores, the more severe the mania is. As a cutoff, a score of 20 is often adopted, but it is arbitrary.

Anxiety was assessed using the Hamilton Anxiety Rating Scale (HARS) [21], one of the first rating scales to measure the severity of perceived anxiety symptoms. It consists of 14 symptom-defined items such as tension, fears, insomnia, cognitive difficulties, depressed mood, and a whole list of somatic symptoms regarding sensory, cardiovascular, respiratory, gastrointestinal, genitourinary, and autonomic systems. Each item is scored on a basic numeric scoring of 0 (not present) to 4 (severe). The higher the score, the more severe the anxiety symptoms are. Scores of 0–7 identify no or minimal anxiety, 8–14 is mild anxiety, 15–23 is moderate anxiety, and ≥24 is severe anxiety.

The **Kessler-10 Psychological Distress Scale** (K-10) [22] is a 10-item self-rated questionnaire intended to assess the amount of distress experienced in the most recent 4-week period. It was used to estimate the psychological impact of COVID-19-related illness. We used the validated Italian translation, which showed high internal consistency reliability, as assessed by Cronbach’s alpha (α = 0.90), with item-total correlations ranging from 0.44 to 0.77 [23]. Each item is scored 1–5 on a Likert scale, ranging from 1 (None of the time) to 5 (All of the time). Higher scores indicate higher levels of psychological distress. A cutoff score of 20 is assumed to be optimal.

*Resilience* was assessed using *the **Connor-Davidson Resilience scale (CD-RISC)*** [24], a 25-item self-report questionnaire, with a 5-point range of responses as follows: not true at all (0), rarely true (1), sometimes true (2), often true (3), and true nearly all of the time (4). The scale is rated based on how the responder has felt over the past month. The total score ranges from 0 to 100, with higher scores reflecting greater resilience. CD-RISC is a unidimensional scale that allows for efficient measurement of an individual’s resilience. Examples of some of the domains in the scale are the ability to cope with stress, the ability to adapt to change, and the ability to stay focused and think clearly.

All psychometric assessments were made by the same clinicians (DJ, AS, SM, BT, AC, LDM, GFF, and GS) who have shown strong interrater reliability for the various psychometric tools used (Cohen’s *kappa* = 0.92 for the HDRS, 0.79 for the HARS, and 0.83 for the YMRS).

### 2.3. Statistical Analyses

For the aim of this study, we subdivided our sample according to gender. We compared the two groups on sociodemographic and clinical characteristics, based on the chi-squared test for nominal variables and one-way analysis of variance (ANOVA) for continuous variables.

We then focused on the distribution patterns of resilience and psychiatric symptoms, including psychic and somatic depressive symptoms, manic symptoms, anxiety, and psychological distress. Therefore, we conducted a multivariate analysis of covariance (MANCOVA) using psychiatric symptoms and resilience as dependent variables and gender as an independent factor. Significant variables between the two groups in the univariate analyses were inserted as covariates to control for the statistical model, where appropriate. When the initial model was significant, we conducted a series of one-way analyses of covariance (ANCOVA) to test differences between groups on dependent variables with the same covariates. We used a statistical model corrected for multiple comparisons according to the Bonferroni procedure (*p* < 0.05/number of comparisons) to minimize the likelihood of type I statistical errors.

For the secondary aim of the study, we also tested the relationship between resilience factors and the risk of psychopathology. Accordingly, linear regression was used to test if resilience significantly predicted psychological distress in the total sample and separately in men and women. For all statistical analyses, we used the Statistical Package for the Social Sciences (SPSS), version 24.0 for Windows (IBM Co., Armonk, NY, USA, 2016).

## 3. Results

A total of 348 individuals were contacted and included in the study. There were no patients unable to provide free, informed consent or who did not know sufficient Italian to complete the questionnaires. Demographic, epidemiological, and clinical characteristics are reported in Table 1.

Of the total sample of patients, 214 were men (61.5%) and 134 (38.5%) were women. Men and women significantly differed in marital status (*χ*^2^ = 25.17, df = 1, *p* < 0.001), living alone (*χ*^2^ = 11.62, df = 1, *p* = 0.01), hospitalization during COVID-19 illness (*χ*^2^ = 12.35, df = 1, *p* < 0.001), and subjective health status before COVID-19 infection (F = 4.32, df = 1, *p* = 0.03) (Table 1). Specifically, most men were married (*n* = 163, 76.2%), not living alone (*n* = 185, 86.4%), were more likely to be hospitalized during COVID-19 illness (*n* = 181, 84.6%), and reported a better subjective health status before COVID-19 infection than women.

### 3.1. Distribution Patterns of Psychiatric Symptoms and Resilience

After controlling for marital status, living alone, hospitalization rate, and subjective health status before COVID-19 infection, psychiatric symptoms and resilience had a significant global effect (Wilks’ Lambda = 0.92, F = 5.03, df = 5, *p* < 0.001) on men and women on the MANCOVA. In particular, a series of ANCOVAs clarified that women significantly reported more psychiatric symptoms than men with respect to psychic and somatic depressive symptoms, anxiety, and psychological distress (Table 2). Women also reported fewer resilience factors than men (Table 2).

### 3.2. Relationship between Resilience Factors and Risk of Psychopathology

**Figure 1:**
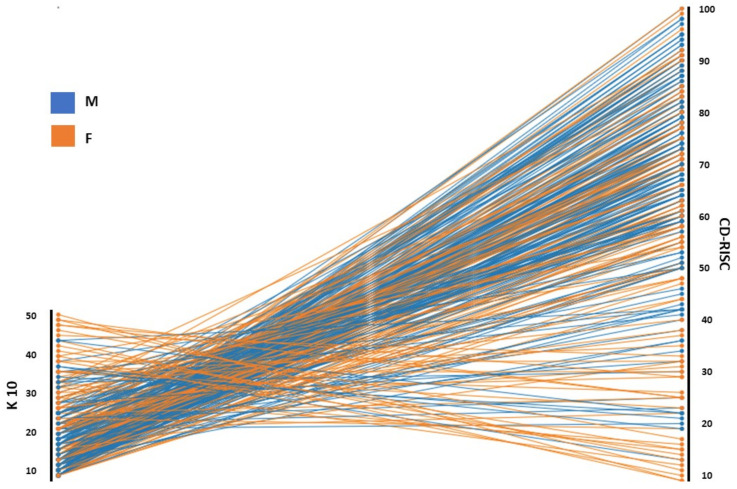
Relationship between resilience factors and risk of psychopathology. Abbreviations: K-10, Kessler Psychological Distress Scale; CD-RISC, Connor-Davidson Resilience Scale; M, men; F, women.

In the total sample, the overall regression was statistically significant (R^2^ = 0.46, *p* < 0.001). In particular, low levels of resilience significantly predicted psychological distress (β = −0.68, *p* < 0.001) (Figure 1). Results were also confirmed in the male (β = −0.62, *p* < 0.001) and female groups, separately considered (β = −0.69, *p* < 0.001).

## 4. Discussion

Our study showed that there were clear gender differences within the sample under investigation in terms of both the sociodemographic characteristics explored, the symptomatology presented, and the response strategies deployed following COVID-19 infection. In particular, we found that perceived subjective health status was already worse prior to COVID-19 illness in the female population, which consisted mostly of unmarried women living alone. Above all, we found a marked predominance of psychiatric symptoms in the elderly female population compared to the male population in the same age group. More specifically, women were found to suffer more from depressive symptoms, both psychological and somatic; they noted anxiety more frequently and scored significantly higher on the general assessment of psychological distress.

These data are consistent with what is available in the literature about the higher prevalence of mood disorders [25,26] and anxiety disorders [27,28] in the elderly female population. Results are also in line with previous studies in the general population, showing higher rates of distress due to the COVID-19 pandemic and associated restriction measures in females compared to males [29,30,31]. According to our results, this observation seems to be confirmed in patients reporting psychiatric symptoms after COVID-19 illness. This holds true despite the majority of hospitalizations and, therefore, more severe forms of COVID-19 illness concern the male gender. Our data may confirm the hypothesis that being a woman represents an independent risk factor for the development of psychological symptoms following infection.

We can make several hypotheses that could explain this sharp gender difference in the experienced symptoms. One possible interpretation is that the elderly female population, although not suffering from the most serious forms of the illness, is more affected by its psychological consequences due, for example, to prolonged isolation in conditions of loneliness. This is the case in the majority of our sample (Table 1), where they were living alone because they were unmarried or widowed, a case also addressed in other studies [32,33]. Another possible factor could be concern about the health status of the partner, who may be hospitalized or undergoing intensive care, and the impossibility to access care facilities, which could have fueled feelings of hopelessness and frustration, as well as the emergence of underlying anxious characteristics. The currently established biological differences in the development of depressive disorders in the two genders, such as higher levels of inflammatory and neurotrophic markers in depressed women [34] and the potential role of sex hormones [35], should also be considered and might account for the observed differences. Further studies are needed to confirm these initial speculations.

Among the possible explanations, we should also take into account a lower tendency of masking symptoms by the female population, especially in old age, compared to men, which could affect the occurrence of anxious and depressive symptoms [36]. Probably, the most coherent explanation is the one that considers the many facets of what is hypothesized so far, and that makes us reflect on the importance of an approach that accounts for gender differences [37]. Accordingly, there are many suggestions that are moving in this direction in recent years, such as the gender balance requirement included for clinical trials [38].

Considering gender differences will also allow us to develop gender-specific units and treatments for this segment of the population, according to the principles of personalized medicine. Moreover, as far as the elderly group is concerned, we have to take into consideration additional factors that characterize women during this period of life, including changes in their hormonal status and lifestyle habits, such as physical activity and nutrition [39]. All these factors may contribute to complicating the psychological status of elderly women and needs special attention [40].

Another interesting finding that emerged from our study and that may also provide therapeutic indications is the role of resilience. Other studies have investigated its importance as related to the pandemic phenomenon, both in health workers [41] and in cohorts of patients. In a recent study, for example, depressive symptoms were found to predict low resilience [42]. In our study, we inverted the possible causal relationship, finding that baseline resilience could be protective against psychological distress and also finding that women scored lower on the resilience scale (Table 2). Multiple regression analyses on both the total sample and the two separate groups confirmed this hypothesis.

The more resilience resources, such as management of negative emotions, acceptance of change, tenacity, and self-confidence are present and functioning, the less a person will be at risk of developing psychological distress [43], even in situations with a high psychological impact, such as COVID-19. Looking at the results on the individual groups, a difference between the two genders emerged in this case where, once again, it was women who paid the price of greater vulnerability. The direct consequence of these results is the need to work much more on the strengthening of these systems, even in those who do not present clear psychopathology, so to protect them from it, for example, by setting up support services aimed at the implementation of resilience strategies.

Before presenting our conclusions, we must acknowledge some issues that might limit the generalizability of our results, such as the relatively small sample and the lack of healthy control groups. In addition, the cross-sectional model of the study does not take into account the great variability over time of this type of symptomatology, nor does it establish cause and effect relationships. A longitudinal design would have been more apt for such a purpose. Furthermore, additional clinical variables may have influenced psychiatric symptoms and resilience levels. 

## 5. Conclusions and Future Directions

The female elderly population affected by COVID-19 showed a greater fragility and vulnerability to psychiatric symptoms after recovering from COVID-19. Elderly women showed less resilience and reported poorer subjective well-being. Our data point to the need to strengthen resilience resources, especially in women. In fact, we will have to act on resilience systems to minimize the development of symptoms in at-risk persons, such as elderly women during (and after) their COVID-19 infection. The enforcement of resilience-enhancing psychotherapeutic techniques could occur if not during the hospitalization or acute phases, at least sometime after having survived COVID-19. Such techniques may incorporate brain plasticity concepts [44]. Psychotherapeutic interventions designed to help patients build resilience (grit, active coping/problem-solving, accommodative coping, and spirituality) may prove effective in preventing and treating depression and other psychiatric symptoms related to the pandemic [45]. Despite resilience being long-considered as a stable trait, it is a multifactorial construct that may respond to treatment at later age [46]. Future studies should investigate the effects of such therapies diachronically, and not only from the post-COVID-19 perspective alone.

## Figures and Tables

**Table 1:** Sociodemographic and clinical characteristics of the sample (*n* = 348) according to gender.

	Men (*n* = 214)	Women (*n* = 134)	F or *χ*2	df	*p*
Age (years): mean ± (SD)	73.25 (6.04)	72.69 (6.43)	0.69	1	0.407
Lives alone: *n* (%)	29 (13.6)	38 (28.4%)	11.62	1	0.001 **
Physical activity: *n* (%)	111 (51.9)	62 (46.3)	1.03	1	0.309
Occupational status, retired: *n* (%)	167 (78.0)	99 (73.9)	0.79	1	0.374
Married: *n* (%)	163 (76.2)	67 (50.0)	25.18	1	<0.001 ***
Education (years): mean ± (SD)	13.10 (5.10)	12.16 (7.66)	1.78	1	0.184
Subjective health status: mean ± (SD)	82.09 (12.18)	79.13 (12.62)	4.32	1	0.038 *
Hospitalization: *n* (%)	181 (84.6)	92 (68.7)	12.36	1	<0.001 ***

* *p* < 0.05; ** *p* < 0.01; *** *p* < 0.001; df, degrees of freedom; F, analysis of variance value (variation between sample means/within sample variation); *P*, significance level; SD, standard deviation.

**Table 2:** Variables resulting from the psychometric assessment of the sample (*n* = 348), according to gender.

	Men (*n* = 214)	Women (*n* = 134)	F	df	*p*
HARS: mean ± (SD)	2.54 (3.54)	5.42 (5.47)	9.35	5	<0.001 ***
HDRS: mean ± (SD)	0.69 (1.42)	1.62 (2.13)	7.28	5	<0.001 ***
HDRS, somatic: mean ± (SD)	1.39 (2.07)	2.53 (2.78)	6.72	5	<0.001 ***
YMRS: mean ± (SD)	0.69 (1.80)	1.16 (2.05)	1.68	5	0.138
K-10: mean ± (SD)	14.74 (5.27)	18.74 (7.91)	8.98	5	<0.001 ***
CD-RISC: mean ± (SD)	69.65 (16.33)	59.76 (21.94)	5.94	5	<0.001 ***

*** *p* < 0.001; df, degrees of freedom; F, analysis of variance value (variation between sample means/within-sample variation); *P*, significance level; SD, standard deviation; HARS, Hamilton Anxiety Rating Scale; HDRS, Hamilton Depression Rating Scale; K-10, Kessler Psychological Distress Scale; YMRS, Young Mania Rating Scale; CD-RISC, Connor-Davidson Resilience Scale.

## Data Availability

Data will be made available anonymously upon any reasonable request to the corresponding author.
